# Host Genetics of Response to Porcine Reproductive and Respiratory Syndrome in Sows: Antibody Response as an Indicator Trait for Improved Reproductive Performance

**DOI:** 10.3389/fgene.2021.707873

**Published:** 2021-08-04

**Authors:** Felipe M. W. Hickmann, José Braccini Neto, Luke M. Kramer, Yijian Huang, Kent A. Gray, Jack C. M. Dekkers, Leticia P. Sanglard, Nick V. L. Serão

**Affiliations:** ^1^Department of Animal Science, Iowa State University, Ames, IA, United States; ^2^Department of Animal Science, Universidade Federal do Rio Grande do Sul, Porto Alegre, Brazil; ^3^Smithfield Premium Genetics, Rose Hill, NC, United States

**Keywords:** Genomics, GWAS, swine, QTL, outbreak, reproduction, PRRS, S/P ratio

## Abstract

Antibody response to porcine reproductive and respiratory syndrome (PRRS) virus (PRRSV) infection, measured as sample-to-positive (S/P) ratio, has been proposed as an indicator trait for improved reproductive performance during a PRRS outbreak in Landrace sows. However, this result has not yet been validated in Landrace sows or evaluated in terminal sire lines. The main objectives of this work were to validate the use of S/P ratio as an indicator trait to select pigs during a PRRS outbreak and to explore the genetic basis of antibody response to PRRSV. Farrowing data included 2,546 and 2,522 litters from 894 Duroc and 813 Landrace sows, respectively, split into pre-PRRS, PRRS, and post-PRRS phases. Blood samples were taken from 1,231 purebred sows (541 Landrace and 690 Duroc) following a PRRS outbreak for subsequent PRRSV ELISA analysis for S/P ratio measurement. All animals had high-density genotype data available (29,799 single nucleotide polymorphisms; SNPs). Genetic parameters and genome-wide association studies (GWAS) for S/P ratio were performed for each breed separately. Heritability estimates (± standard error) of S/P ratio during the PRRS outbreak were moderate, with 0.35 ± 0.08 for Duroc and 0.34 ± 0.09 for Landrace. During the PRRS outbreak, favorable genetic correlations of S/P ratio with the number of piglets born alive (0.61 ± 0.34), number of piglets born dead (−0.33 ± 0.32), and number of stillborn piglets (−0.27 ± 0.31) were observed for Landrace sows. For Duroc, the GWAS identified a major quantitative trait locus (QTL) on chromosome (Chr) 7 (24-15 megabases; Mb) explaining 15% of the total genetic variance accounted for by markers (TGVM), and another one on Chr 8 (25 Mb) explaining 2.4% of TGVM. For Landrace, QTL on Chr 7 (24–25 Mb) and Chr 7 (108–109 Mb), explaining 31% and 2.2% of TGVM, respectively, were identified. Some of the SNPs identified in these regions for S/P ratio were associated with reproductive performance but not during the PRRS outbreak. Genomic prediction accuracies for S/P ratio were moderate to high for the within-breed analysis. For the between-breed analysis, these were overall low. These results further support the use of S/P ratio as an indicator trait for improved reproductive performance during a PRRS outbreak in Landrace sows.

## Introduction

Recent studies have shown that reproductive performance traits in commercial sows infected with porcine reproductive and respiratory syndrome (PRRS) virus (PRRSV) have low heritability estimates (Rashidi et al., 2014; Serão et al., 2014; Putz et al., 2019; Scanlan et al., 2019; Hickmann et al., 2021). Therefore, identifying an indicator trait highly heritable and highly genetically correlated with reproductive performance under a PRRS outbreak could be used to select for reproductive performance in PRRSV-infected sows.

Antibody response to PRRSV infection, measured as sample-to-positive (S/P) ratio using a commercial ELISA, has been proposed as an indicator trait to improve litter size traits in sows infected with PRRSV. Serão et al. (2014) reported a moderately high estimate of heritability for S/P ratio (0.45) measured approximately 46 days after the beginning of the PRRS outbreak. These authors also estimated large genetic correlations of S/P ratio with the number of piglets born alive (NBA; 0.73) and stillborn piglets (NSB; −0.72) during a PRRS outbreak. These results indicate that selection for increased S/P ratio would result in a correlated response to selection in NBA and NSB under PRRS that is 63% and 97% more efficient than direct selection for NBA and NSB, respectively. The high heritability of S/P ratio has been validated by Serão et al. (2016). In contrast, Putz et al. (2019) reported a relatively low heritability estimate (0.17) for S/P ratio measured at approximately 60 days after the predicted start of the PRRS outbreak. However, the method of measuring S/P ratio in their study was not the same as in others (Serão et al., 2014, 2016; Abella et al., 2019; Sanglard et al., 2020). Nonetheless, Putz et al. (2019) found a high genetic correlation of S/P ratio with NSB under PRRS (−0.73), supporting the findings of Serão et al. (2014).

Genomic analyses for S/P ratio following a PRRSV infection are scarce in the literature, with Serão et al. (2014, 2016) being the only studies that have reported QTL for this trait in PRRSV-infected sows. Serão et al. (2014) identified two major QTL on *Sus scrofa* chromosome (SSC) 7 that together explained 40% of the total genetic variance accounted for by markers (TGVM) for S/P ratio. The two QTL identified by Serão et al. (2014) were further validated by Serão et al. (2016). One of these QTL explained 25% of the TGVM and was located in the Major Histocompatibility Complex (MHC) region, a gene-rich region in the genome that harbors several genes playing essential roles in the immune system of mammals (Hammer et al., 2020). In addition, Sanglard et al. (2020) also identified the MHC QTL in gilts vaccinated with a commercial modified live virus vaccine. In addition, Serão et al. (2014, 2016) also identified specific single nucleotide polymorphisms (SNPs) associated with S/P ratio, indicating that key SNPs can be used to select for this trait.

Serão et al. (2016) reported moderate genomic prediction accuracies for S/P ratio in commercial gilts. This indicates that phenotypic and genomic information collected at the commercial level can be used to estimate marker effects accurately and breeding values for nucleus herds to genetically select animals with increased S/P ratio when exposed to PRRSV. Although S/P ratio has potential as an indicator trait for genetic improvement of litter size traits in PRRSV-infected sows, the high genetic correlation between these traits and S/P ratio reported by Serão et al. (2014) requires validation in other datasets and breeds. Therefore, the main objectives of this work were to validate the use of S/P ratio as an indicator trait for improved reproductive performance during a PRRS outbreak, to perform genomic analyses of S/P ratio, and to evaluate the effects of key SNPs on S/P ratio and reproductive performance in Landrace and Duroc sows.

## Materials and Methods

All animal experimental procedures used in this study were followed according to international guidelines on Animal Care under industry standard conditions (IACUC, Iowa State University, protocol number 6-17-8551-S).

### Source of Data

The data used in this study were obtained from two commercial purebred herds (Duroc and Landrace) that experienced a PRRS outbreak during the spring of 2018. The PRRS outbreak was identified based on a combination of previous methodologies (Lewis et al., 2009; Putz et al., 2019; Scanlan et al., 2019), as described by Hickmann et al. (2021). The wild-type PRRSV strain was sequenced and identified as PRRSV 1-7-4, a highly pathogenic strain. The focus of the study performed by Hickmann et al. (2021) was on the genomic basis of reproductive performance in healthy and PRRSV-infected sows. In contrast, this study focuses on the genomic basis of S/P ratio and its relationship with reproductive performance in healthy and PRRSV-infected sows. Briefly, the farrowing data included 2,546 and 2,522 litters from 894 Duroc and 813 Landrace sows, respectively, split into pre-PRRS, PRRS, and post-PRRS phases. The number of animals (litters) included in the pre-PRRS, PRRS, and post-PRRS datasets were 478 (1,004), 501 (501), and 558 (1,079), respectively, for Duroc, and 461 (1,096), 429 (429), and 527 (1,025), respectively, for Landrace. Not all animals experienced all three PRRS phases. The reproductive data included farrowing performance for the number of piglets born alive (NBA, pigs/litter), number of stillborn piglets (NSB, pigs/litter), number of mummified piglets (NBM, pigs/litter), number of piglets born dead (NBD, pigs/litter; the sum of NSB and NBM), total number of piglets born (TNB, pigs/litter; the sum of NBA and NBD), and number of piglets weaned (NW, piglets/litter). The net number of cross-fostered piglets (fostered in minus fostered out) was also available. A total of 450 (23%) Duroc and 433 (23%) Landrace litters had cross-fostering. Prior to analyses, NSB, NBM, and NBD were transformed as ln(phenotype+1), following Serão et al. (2014), because of the right skewness observed in the data. Sows from Duroc and Landrace herds originated from 71 sires and 446 dams, and 92 sires and 365 dams, respectively.

Following the appearance of typical signs of PRRSV infection, such as changes in reproductive performance and clinical symptoms, sows on the farm were bled using Lavender Top Vacutainer tubes (Becton, Dickinson and Company, Franklin Lakes, NJ, United States) on June 1^st^, 2018, approximately 54 days after the detection of PRRS (Hickmann et al., 2021). Blood samples were shipped to the Veterinary Diagnostic Laboratory at Iowa State University (Ames, IA, United States), where sera from these samples were used to quantify PRRSV antibody by ELISA (PRRS X3 Ab Test, IDEXX, Westbrook, ME, United States). The PRRSV antibody assay produced a quantitative result (i.e., S/P ratio) of the adjusted sample optical density (OD) divided by the adjusted kit positive control serum OD. These OD adjustments subtracted the average negative control OD from the sample OD and the average positive control OD. The S/P ratio data consisted of 690 Duroc and 541 Landrace sows. Of these, 644 Duroc and 528 Landrace sows also had reproductive data available.

All animals had follicular hair or ear tissue samples taken and shipped to Neogen GeneSeek (Lincoln, NE, United States) for genotyping using the GGP Porcine HD panel (Neogen GeneSeek) for 45,536 SNPs. The genotype data were processed according to the breeding company’s pipeline, including the removal of non-segregating SNPs, SNPs with a minor allele frequency of less than 0.05, and minimum SNP call rate and animal call rate of 0.9. In addition, missing genotypes were imputed using Fimpute 2.2 (Sargolzaei et al., 2014). These steps were performed within bread, resulting in 29,799 SNPs common to both breeds used in the final analyses, with the *Sscrofa* 11.1 assembly being used for the SNP location.

### Genetic Parameters

Heritability estimates of S/P ratio were estimated using a univariate model, while correlations of S/P ratio with reproductive traits were estimated using a bivariate model. A genomic relationship matrix (***GRM***) was derived for each breed separately based on VanRaden (2008), method 1. Analyses were performed for each breed separately. Heritability estimates of S/P ratio were obtained from the following univariate animal model:

(1) $$Y_{i\,j}=\mu +P\,A\,R_{i}+a_{j}+e_{i\,j}$$

where *Y*_ij_ is the observed phenotype; μ is the general mean; *PAR*_*i*_ is the fixed-effect of the *i*^th^ parity (*i* = 1,…6); *a_j* is the animal random effect, assuming $a_{j}\,N\,\left(0,\text{GRM}\,\sigma_{a}^{2}\right)$, where $\sigma_{a}^{2}$ is the additive genetic variance; and *e*_ij_ is the random residual term associated with *Y*_ij_, assuming $e_{\text{ij}}\,N\,\left(0,\text{I}\,\sigma_{e}^{2}\right)$, where **I** is the identity matrix and $\sigma_{e}^{2}$ is the residual variance. Heritability estimates of reproductive performance traits were obtained from a bivariate model with S/P ratio. The bivariate model used included the same effects as the univariate model for each trait. The models used for reproductive traits are described in Hickmann et al. (2021). All analyses were performed in ASReml 4.0 (Gilmour et al., 2015).

### Genome-Wide Association Studies

Genome-wide association studies (GWAS) were performed for S/P ratio for each breed separately using the BayesB method (Habier et al., 2011). In the GWAS, we used π = 0.9973 to fairly compare our results with those reported by Serão et al. (2014), i.e., including the same expected number of SNPs in the model (80 SNPs). The GWAS model included the fixed effects of intercept and parity, and the random allele substitution effects for the SNP markers. For all analyses, additive genetic and residual variances from the genetic parameter analyses were used as priors. A total of 50,000 Markov Chain Monte Carlo (MCMC) iterations were used, with 10,000 iterations used as burn-ins and MCMC samples stored at every 100^th^ iteration, resulting in 500 posterior MCMC samples. Results from this analysis are presented as the posterior mean of the %TGVM of non-overlapping 1-Mb regions based on the *Sscrofa11.1* genome build. Genomic regions explaining ≥ 2 %TGVM were considered significant. For a given 1-Mb region *i* including *m* SNPs, its %TGVM was calculated as:

(2) %TGVM=i∑k=1500var(∑j=1nM⁢αi⁢ki⁢j′∑i=1w∑j=1nM⁢αi⁢ki⁢j′)k

where ***%TGVM***_i_ represents the vector of calculated ***%TGVM*** of the *i*^th^ 1-Mb region including *m* SNPs; ***M***${}_{\text{ij}}^{\prime }$ represents the row vector of reference allele counts (coded −1, 0, 1) of the *m* SNPs included in the *i*^th^ 1-Mb region of the genome for the *j*^th^ animal (*j* = 1 to *n*, where *n* changed across breeds and traits); and *α*_ik_ represents the column vector of allele-substitution effects of the *m* SNPs included in the *i*^th^ 1-Mb region of the genome in the *k*^th^ stored MCMC sample. For both breeds, the number *w* of 1-Mb non-overlapping windows was 2,211 and the average (SD) number *m* of SNPs within each 1-Mb region was 13.5 (8).

Before final results, 1-Mb windows showing %TGVM ≥ 0.5% within 3 Mb from significant regions (i.e., ≥2 %TGVM) were combined into one larger window and its %TGVM was recalculated as described in Eq. (2). This strategy was used due to the resolution of Bayesian GWAS methods, where simulations have shown that quantitative loci nucleotides are expected to be located within 3 Mb of the identified QTL regions (Garrick and Fernando, 2013). For the presentation of GWAS results in figures and tables, the start of the QTL region on a given SSC *c* was assumed to be *c:Mb_i_,000,000*, and the end of the QTL region as *c:Mb_f_,999,999* where *Mb*_i_ and *Mb*_f_ represent the Mb where the identified QTL window started and ended, respectively. Thus, for example, if a QTL was identified in a given 1-Mb region *r*, the position of the QTL was expressed as *rMb*, such that *Mb*_i_ = *Mb*_f_ = *r* and the QTL encompassed *c:r,000,000–r,999,999*. In contrast, when closely located 1-Mb QTL regions were combined into a single window, the position of the QTL was expressed as *r-r’Mb*, such that *Mb*_i_ = *r* < *Mb*_f_ = *r’* and the QTL encompassed *c:r,000,000–r’,999,999*. All analyses were performed using GenSel version 4.4 (Fernando and Garrick, 2009). The linkage disequilibrium (LD) among SNPs in identified QTL regions for S/P ratio was estimated using the Haploview software (Barrett, 2009).

### Effect of Selected SNPs Associated With S/P Ratio

We investigated the effects of SNPs identified to be associated with S/P ratio in this study and by Serão et al. (2014). Based on results from this study, SNPs showing the largest estimated allele substitution effects within identified QTL in the GWAS for S/P ratio were further evaluated. SNPs selected based on the study of Serão et al. (2014) were ASGA0031860 (7:22,075,114), MARC0058875 (7:24,865,378), and ASGA0032151 (7:25,967,157), based on the *Sscrofa11.1* genome build. Although two additional SNPs were also associated with S/P ratio in Serão et al. (2014), these SNPs were not present in the genotype data in this study. The effect of these selected SNPs on S/P ratio and reproductive traits was tested with separate analyses for each breed and PRRS phase by simultaneously fitting all selected SNPs as categorical fixed effects in the model used for estimation of genetic parameters. Significance of the effect of a SNP was declared at a *p*-value < 0.05, and a trend was declared at 0.05 < *p*-value < 0.10. Analyses were performed using ASReml 4.0.

### Genomic Prediction of S/P Ratio

Genomic prediction analyses for S/P ratio were performed with the model described for GWAS, using BayesB (π = 0.9973) in GenSel v.4.4 (Fernando and Garrick, 2009). Analyses were performed both within-breed and between breeds. For the within-breed analysis, 5-fold cross-validation was performed for each breed separately, where each animal was randomly assigned to one of five folds. In this approach, markers were trained using four folds and validated using the remaining fold. This was repeated five times until all validation datasets were used for validation. There were 138 and 108 animals in each fold for Duroc and Landrace, respectively (one fold for Landrace had 109 observations). Genomic prediction accuracies (GPAs) were calculated as a weighted average as:

(3) $$G\,P\,A=\frac{\sum_{i=1}^{5}n_{i}\,r_{i}\,\left(G\,E\,B\,V,y^{*}\right)}{\sum_{i=1}^{5}n_{i}\,\sqrt{h^{2}}}$$

where *r*_*i*_(*GEBV*,*y*^∗^) is the correlation between the genomic estimated breeding value (GEBV) and phenotypes adjusted for fixed effects (*y*^∗^) in the *i*^th^ validation dataset, weighted by the number of records in each validation dataset (*n*_*i*_); and *h*^2^ is the heritability of S/P ratio for the whole dataset of the breed being analyzed.

For the between-breed analysis, all data for one breed were used as training to validate the other breed. Thus, GPA was calculated as the correlation between GEBV and *y*^∗^, divided by the square root of the estimate of heritability based on the whole dataset for the breed used for validation.

Both the within- and between-breed analyses were performed using three sets of SNPs, as proposed by Serão et al. (2016):

• All 29,799 SNPs across the genome (SNP_All_);
• Only SNPs in the QTL that harbors the MHC region (SNP_MHC_);
• All SNPs across the genome, excluding those in the MHC region (SNP_Rest_).

The SNPs in the MHC region were defined based on the GWAS results of each breed. For SNP_Rest_, a 2-Mb window surrounding the QTL found in the MHC region was removed to avoid having any SNPs in LD with this QTL that could affect results.

## Results

### Genetic Parameters

The estimate of heritability of S/P ratio was moderately high for both breeds (Table 1). For Duroc, the estimate (0.35 ± 0.08) was numerically greater than for Landrace (0.34 ± 0.09). In general, variance component estimates were very similar between breeds. Landrace sows had a numerically greater additive genetic variance estimate (0.033) than Duroc sows (0.032). On the other hand, the residual variance estimate (0.059) was slightly lower for Duroc than for Landrace (0.063).

**TABLE 1:** Heritability estimates (SE) and variance components of traits and their genetic correlation (*r_g*) with sample-to-positive (S/P) ratio for each PRRS phase and breed.

Trait	Duroc				Landrace			
	h^2^ (SE)	$\sigma_{a}^{2}$	$\sigma_{e}^{2}$	r_g_ (SE)	h^2^ (SE)	$\sigma_{a}^{2}$	$\sigma_{e}^{2}$	r_g_ (SE)
**Pre-PRRS**								

TNB	0.14 (0.04)	1.08	6.51	0.04 (0.19)	0.11 (0.03)	1.64	13.36	0.12 (0.21)
NBA	0.19 (0.04)	1.35	5.68	−0.04 (0.18)	0.13 (0.03)	1.42	9.86	0.15 (0.20)
NBD	0.01 (0.02)	0.01	1.47	NC	0.06 (0.03)	0.02	0.35	−0.18 (0.25)
NSB	0.05 (0.03)	0.01	0.21	0.48 (0.24)	0.10 (0.03)	0.03	0.26	−0.13 (0.21)
NBM	0.03 (0.02)	<0.01	0.13	−0.38 (0.31)	0.01 (0.02)	<0.01	0.18	0.01 (0.47)
NW	0.09 (0.03)	0.34	3.60	−0.34 (0.22)	0.07 (0.03)	0.46	5.83	0.05 (0.25)

**PRRS**								

S/P ratio	0.35 (0.08)	0.032	0.059	–	0.34 (0.09)	0.033	0.063	–
TNB	0.12 (0.07)	1.15	8.27	0.23 (0.27)	0.01 (0.05)	0.14	15.25	0.47 (1.47)
NBA	0.11 (0.07)	1.20	9.67	−0.24 (0.30)	0.08 (0.07)	1.77	20.53	0.61 (0.34)
NBD	0.08 (0.06)	0.05	0.54	NC	0.12 (0.07)	0.10	0.71	−0.33 (0.32)
NSB	0.02 (0.05)	0.01	0.30	0.01 (0.54)	0.14 (0.08)	0.06	0.35	−0.27 (0.31)
NBM	0.03 (0.05)	<0.01	0.53	−0.08 (0.41)	0.08 (0.07)	0.07	0.84	−0.11 (0.37)
NW	0.12 (0.06)	1.28	9.00	0.30 (0.25)	0.08 (0.07)	1.40	15.86	0.10 (0.37)

**Post-PRRS**								

TNB	0.15 (0.04)	1.37	7.58	−0.22 (0.17)	0.20 (0.04)	2.71	10.95	−0.06 (0.17)
NBA	0.13 (0.04)	0.98	6.79	−0.17 (0.18)	0.16 (0.04)	1.87	9.98	0.06 (0.18)
NBD	0.12 (0.04)	0.17	1.17	NC	0.10 (0.03)	0.04	0.33	−0.26 (0.20)
NSB	0.07 (0.03)	0.01	0.18	−0.04 (0.22)	0.12 (0.03)	0.03	0.23	−0.20 (0.19)
NBM	0.06 (0.03)	0.01	0.11	−0.17 (0.23)	0.01 (0.02)	<0.01	0.19	−0.33 (0.54)
NW	0.14 (0.04)	1.12	6.77	−0.11 (0.18)	0.14 (0.04)	1.45	8.78	−0.09 (0.20)

**S/P ratio*, sample-to-positive ratio; *TNB*, total number of piglets born; *NBA*, number of piglets born alive; *NBD*, number of piglets born dead; *NSB*, number of stillborn piglets; *NBM*, number of mummified piglets; *NW*, number of piglets weaned; *NC*, not converged.*

Heritability estimates of reproductive traits and genetic correlations (*r*_g_) of S/P ratio with farrowing traits are shown in Table 1. Only results that converged are shown. Genetic correlation estimates of reproductive traits with S/P ratio in Duroc sows were moderate to low, ranging from −0.38 ± 0.31 (NBM) to 0.48 ± 0.24 (NSB) for pre-PRRS, −0.24 ± 0.30 (NBA) to 0.30 ± 0.25 (NW) for PRRS, and −0.22 ± 0.17 (NBA) to −0.04 ± 0.22 (NSB) for post-PRRS. For Landrace, these were also moderate to low and ranged from −0.18 ± 0.25 (NBD) to 0.15 ± 0.20 (NBA) for pre-PRRS, −0.33 ± 0.32 (NBD) to 0.61 ± 0.34 (NBA) for PRRS, and −0.33 ± 0.54 (NBM) to 0.06 ± 0.18 (NBA) for post-PRRS. Pre-PRRS and PRRS in Landrace sows showed favorable genetic correlation estimates of S/P ratio with reproductive performance, whereas for Duroc, these relationships were not strong.

### Genomic Regions in the Pig Genome Associated With S/P Ratio

**FIGURE 1:**
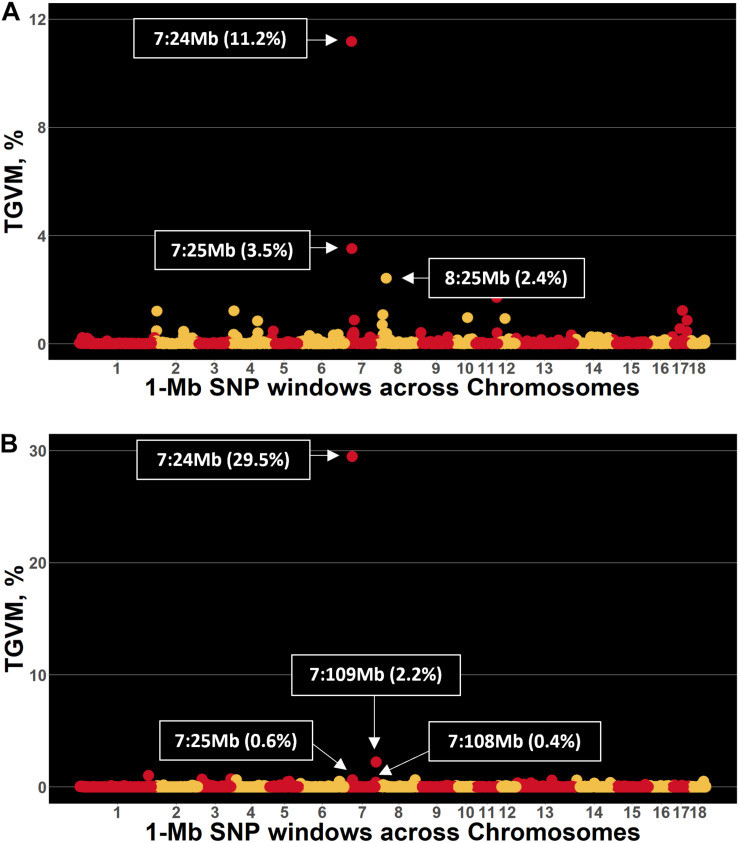
Manhattan plot for sample-to-positive (S/P) ratio during the porcine reproductive and respiratory syndrome (PRRS) outbreak in Duroc and Landrace sows. Each point represents a 1-Mb SNP window (*x*-axis) plotted against the percentage of total genetic variance accounted for by markers (TGVM; *y*-axis). **(A,B)** Results for Duroc and Landrace sows, respectively.

**FIGURE 2:**
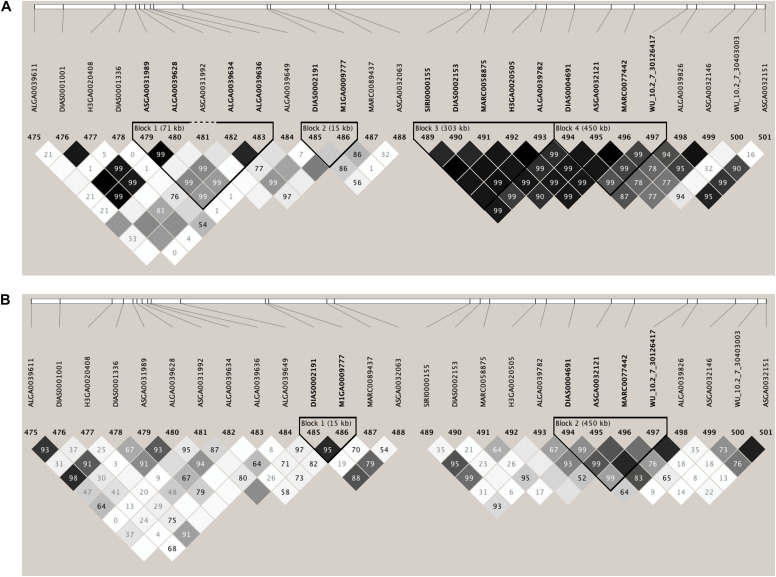
Linkage disequilibrium (LD) plots of the genotype data for the 3-Mb SNP window that harbors the major histocompatibility complex (MHC) on *Sus Scrofa* chromosome 7 (SSC 7: 24–25 Mb) associated with sample-to-positive (S/P) ratio. **(A,B)** Results for Duroc and Landrace sows, respectively. LD is expressed as *r*^2^. The darker diamonds indicate greater LD. These plots indicate lower LD in Landrace sows than Duroc sows within this region.

Few genomic regions that explained a substantial proportion of TGVM were identified in Duroc and Landrace sows for S/P ratio (Figure 1). For Duroc, two 1-Mb regions within 1 Mb from each other on SSC 7 explained 11.2% (24 Mb; i.e., 7:24,000,000:24,999,999) and 3.5% (25 Mb) of TGVM, and one on SSC8 (25 Mb) that explained 2.4% of TGVM were identified (Figure 1A). Once the two regions on MHC region were combined and its %TGVM recalculated, the QTL (24–25 Mb; i.e., 7:24,000,000–25,999,999) explained 15% of TGVM. For Landrace, four 1-Mb regions on SSC 7 were identified (Figure 1B). Two of these were located within 1 Mb, with one explaining 29.5% of TGVM (24 Mb) and the other 0.6% of TGVM (25 Mb). Once combined, this QTL (24–25 Mb) explained 31% of TGVM. The other two QTL on SSC 7 were also located within 1 Mb of each other, with one explaining 0.5% of TGVM (Mb 108) and the other 2.2% of TGVM (109 Mb). Once combined (108–109 Mb), this QTL explained 2.2% of TGVM. The QTL located on SSC 7 (24–25 Mb) for both breeds harbors the MHC region, the most important genomic region controlling immune response in mammals. We also investigated the LD in this region for each breed (Figure 2). In general, LD in this region was much lower for Landrace (Figure 2B) sows than for Duroc (Figure 2A) sows.

### Effect of Selected SNPs Associated With S/P Ratio

The SNPs within the two QTL regions found for each breed were subjected to additional investigation to identify SNPs with large effects that could be responsible for the %TGVM explained for by these regions. From this, we identified one SNP for Duroc (MARC0089437; 7:24,217,931) and one for Landrace (ASGA0032063; 7:24,247,099), both located on the MHC region. These two SNPs were combined with the three previously reported by Serão et al. (2014), and their associations with S/P ratio were investigated in this study. Results of these associations are presented in Table 2. For Duroc sows, the MARC0089437 SNP explained 9.2% of TGVM, while all other SNPs explained less than 0.3% of TGVM. Of the five selected SNPs, MARC0089437 and MARC0058875 were significantly (*p*-value ≤ 0.018) associated with S/P ratio. For Landrace sows, the ASGA0032063 SNP explained 29.1% of TGVM, while all other SNPs explained less than 0.7% of TGVM. Of the five selected SNPs, ASGA0032063, MARC0089437, and ASGA0032151 were significantly (*p*-value ≤ 0.054) associated with S/P ratio. These results provide information on key SNPs associated with S/P ratio for Duroc and Landrace sows during a PRRS outbreak.

**TABLE 2:** Effect^1^ of selected SNPs^2^ associated with sample-to-positive (S/P) ratio during the PRRS outbreak for each breed.

SNP name	%TGVM^3^	Genotype			MAF	*p*-value
		*AA*	*AB*	*BB*		
*Duroc*						
ASGA0031860	<0.1	1.03 (0.07)	1.09 (0.04)	1.04 (0.03)	0.19 (A)	0.202
MARC0089437	9.2	0.86^b^ (0.07)	1.10^a^ (0.05)	1.21^a^ (0.06)	0.41 (A)	0.002
ASGA0032063	<0.1	1.04 (0.03)	1.07 (0.05)	–	0.05 (B)	0.519
MARC0058875	0.2	1.04^a^ (0.07)	0.97^b^ (0.05)	1.15^a^ (0.06)	0.41 (B)	0.018
ASGA0032151	<0.1	1.08 (0.06)	1.06 (0.04)	1.02 (0.04)	0.36 (A)	0.517
*Landrace*						
ASGA0031860	<0.1	1.51 (0.11)	1.47 (0.10)	1.44 (0.10)	0.35 (A)	0.511
MARC0089437	<0.1	–	1.39^a^ (0.05)	1.28^b^ (0.02)	0.04 (A)	0.034
ASGA0032063	29.1	1.48^b^ (0.12)	1.55^a^ (0.10)	1.39^c^ (0.10)	0.31 (A)	0.002
MARC0058875	<0.1	1.48 (0.10)	1.45 (0.10)	1.49 (0.11)	0.40 (A)	0.617
ASGA0032151	0.6	1.40^b^ (0.10)	1.48^ab^ (0.10)	1.53^a^ (0.10)	0.41 (B)	0.054

**%TGVM*, percentage of the total genetic variance of S/P ratio explained by the marker; *MAF*, minor allele frequency (minor allele in parenthesis); *PRRS*, porcine reproductive and respiratory syndrome.*

*^1^Results for each genotype expressed as expected values for S/P ratio (standard errors within parenthesis), measurement of antibody response to PRRSV.*

*^2^SNPs are located on *Sus scrofa* chromosome 7 as follow according to the *Sscrofa11.1* (GCA_000003025.6) assembly: ASGA0031860/rs80959936 (7:22,075,114), MARC0089437/rs80900036 (7:24,217,931), ASGA0032063/rs80940999 (7:24,247,099), MARC0058875/rs80986722 (7:24,865,378), and ASGA0032151/rs80947467 (7:25,967,157). SNP markers MARC0089437 and ASGA0032063 were associated with S/P ratio in this study, in Duroc and Landrace populations, respectively. The other markers were associated with S/P ratio in Landrace sows in Serão et al. (2014).*

*^3^The remaining of the TGVM of the S/P ratio QTL after accounting for the %TGVM of these SNPs were 1.5% and <0.1%, for Duroc and Landrace, respectively.*

*^*a*–*c*^Expected values within row lacking the same superscript indicate differences at *p*-value <0.05.*

### Effect of Selected SNPs Associated With S/P Ratio on Reproductive Traits

Estimates of the association of the five selected SNPs with reproductive performance are shown in Table 3. Overall, few associations were found to be significant. Interestingly, there were no associations (*p*-value ≥ 0.370) of these selected SNPs with reproductive traits in Landrace sows during the PRRS phase. For both breeds, most associations were found for the post-PRRS period. Starting with pre-PRRS, the MARC0089437 SNP had a trending association with NBA (*p*-value = 0.089) in Duroc sows, with heterozygotes showing greater performance than both homozygotes. In Landrace sows, this SNP had a trending association with NW (*p*-value = 0.063), with *BB* animals having greater NW than *AB* animals, while no *AA* animals were observed in the dataset. The ASGA0032063 SNP had a trending effect (*p*-value ≤ 0.067) for two mortality traits (NBD and NSB) in Landrace sows during pre-PRRS, with *AA* animals showing lower NBD and NSB than animals with the other two genotypes. For the PRRS phase in Duroc sows, the ASGA0031860 SNP had a trending association with NBD (*p*-value = 0.058), with *BB* sows having greater NBD than *AA* and *AB* sows. For the post-PRRS phase, the ASGA0031860 SNP was associated (*p*-value = 0.05) and had a trending effect (*p*-value = 0.09) for NW in Landrace and Duroc sows, respectively, with *AA* animals showing greater performance than the other genotypes for both breeds. The ASGA0032151 SNP also had a trending effect for NW (*p*-value = 0.057) and NSB (*p*-value = 0.089) in Landrace sows only, with *AA* sows showing overall greater performance for both traits. The MARC0089437 SNP was associated with several traits (TNB, NBA, NBM, and NW; *p*-value ≤ 0.068) in Duroc sows in the post-PRRS period. Although *AA* sows had greater NBM than *AB* and *BB* sows, they also had greater TNB, NBA, and NW. However, differences for TNB, NBA, and NW were between *BB* with *AA* and *AB*, indicating that heterozygote animals have overall better reproductive performance than the other genotypes. This same SNP had a trending effect for TNB (*p*-value = 0.081) in Landrace sows, with *AA* sows showing greater performance than *AB* sows. On the other hand, the *AA* genotype of the MARC0058875 SNP in Duroc sows was associated not only with greater TNB (*p*-value = 0.037) and NW (*p*-value = 0.069) but also greater NBM (*p*-value = 0.013) compared with the other genotypes. These results provide additional information on key SNPs associated with S/P ratio in Duroc and Landrace sows during a PRRS outbreak and their associations with reproductive performance across PRRS phases.

**TABLE 3:** Effect^1^ of selected SNPs^2^ associated with sample-to-positive (S/P) ratio on reproductive traits^3^ across PRRS phases.

SNP	Genotypes	TNB	NBA	NBD	NSB	NBM	NW
**Pre-PRRS**							

*Duroc*							
ASGA0031860	*AA*	9.74 (0.68)	8.45 (0.67)	0.78 (0.12)	0.54 (0.11)	0.28 (0.09)	7.29 (0.48)
	*AB*	9.22 (0.34)	8.25 (0.34)	0.64 (0.06)	0.46 (0.06)	0.18 (0.04)	7.21 (0.25)
	*BB*	9.26 (0.27)	8.13 (0.27)	0.74 (0.05)	0.50 (0.04)	0.23 (0.04)	7.11 (0.21)
	*p*-value	0.698	0.822	0.301	0.713	0.310	0.816
MARC0089437	*AA*	9.40 (0.64)	8.31^B^ (0.63)	0.68 (0.12)	0.42 (0.11)	0.27 (0.08)	7.56 (0.46)
	*AB*	9.92 (0.47)	8.79^A^ (0.46)	0.72 (0.08)	0.52 (0.08)	0.20 (0.06)	7.34 (0.34)
	*BB*	8.90 (0.61)	7.73^B^ (0.60)	0.75 (0.11)	0.57 (0.10)	0.21 (0.08)	6.71 (0.43)
	*p*-value	0.107	0.089	0.967	0.803	0.866	0.195
ASGA0032063	*AA*	9.45 (0.31)	8.26 (0.30)	0.77 (0.06)	0.52 (0.05)	0.25 (0.04)	7.13 (0.23)
	*AB*	9.37 (0.47)	8.29 (0.46)	0.67 (0.08)	0.48 (0.08)	0.20 (0.06)	7.28 (0.34)
	*p*-value	0.823	0.931	0.344	0.687	0.349	0.544
MARC0058875	*AA*	9.83 (0.55)	8.75 (0.55)	0.68 (0.10)	0.50 (0.09)	0.19 (0.07)	7.57 (0.40)
	*AB*	9.27 (0.47)	8.20 (0.46)	0.69 (0.09)	0.46 (0.08)	0.24 (0.06)	7.25 (0.34)
	*BB*	9.11 (0.70)	7.88 (0.69)	0.79 (0.12)	0.55 (0.11)	0.26 (0.09)	6.80 (0.50)
	*p*-value	0.523	0.514	0.931	0.842	0.750	0.491
ASGA0032151	*AA*	9.51 (0.53)	8.61 (0.52)	0.57 (0.09)	0.45 (0.08)	0.14 (0.07)	7.35 (0.38)
	*AB*	9.27 (0.39)	8.02 (0.38)	0.81 (0.07)	0.57 (0.06)	0.25 (0.05)	7.09 (0.28)
	*BB*	9.44 (0.37)	8.20 (0.36)	0.79 (0.07)	0.49 (0.06)	0.29 (0.05)	7.17 (0.27)
	*p*-value	0.736	0.302	0.132	0.313	0.152	0.624
*Landrace*							
ASGA0031860	*AA*	13.35 (0.62)	11.91 (0.54)	1.03 (0.09)	0.68 (0.08)	0.28 (0.06)	9.00 (0.39)
	*AB*	13.78 (0.39)	12.18 (0.34)	1.09 (0.05)	0.69 (0.05)	0.34 (0.04)	9.54 (0.25)
	*BB*	13.31 (0.34)	11.91 (0.30)	0.99 (0.05)	0.65 (0.04)	0.29 (0.03)	9.54 (0.23)
	*p*-value	0.332	0.604	0.623	0.877	0.438	0.305
MARC0089437	*AB*	13.33 (0.53)	11.76 (0.46)	1.12 (0.08)	0.70 (0.07)	0.35 (0.05)	9.08^B^ (0.34)
	*BB*	13.61 (0.29)	12.24 (0.26)	0.96 (0.04)	0.65 (0.04)	0.26 (0.03)	9.65^A^ (0.20)
	*p*-value	0.587	0.256	0.291	0.699	0.200	0.063
ASGA0032063	*AA*	13.65 (0.77)	12.63 (0.67)	0.66^B^ (0.11)	0.39^B^ (0.10)	0.20 (0.08)	9.54 (0.49)
	*AB*	13.49 (0.45)	11.79 (0.39)	1.25^A^ (0.06)	0.84^A^ (0.06)	0.34 (0.04)	9.34 (0.29)
	*BB*	13.30 (0.54)	11.58 (0.47)	1.26^A^ (0.08)	0.85^A^ (0.07)	0.38 (0.05)	9.20 (0.34)
	*p*-value	0.925	0.512	0.067	0.061	0.386	0.865
MARC0058875	*AA*	13.24 (0.51)	11.49 (0.44)	1.28 (0.07)	0.84 (0.07)	0.38 (0.05)	9.34 (0.33)
	*AB*	13.50 (0.43)	12.25 (0.38)	0.88 (0.06)	0.57 (0.06)	0.27 (0.04)	9.50 (0.28)
	*BB*	13.70 (0.58)	12.26 (0.51)	0.97 (0.09)	0.63 (0.08)	0.27 (0.06)	9.25 (0.37)
	*p*-value	0.843	0.383	0.110	0.138	0.416	0.648
ASGA0032151	*AA*	13.30 (0.50)	11.87 (0.44)	0.97 (0.07)	0.64 (0.07)	0.29 (0.05)	9.47 (0.32)
	*AB*	13.69 (0.41)	12.22 (0.36)	1.01 (0.06)	0.70 (0.05)	0.26 (0.04)	9.29 (0.27)
	*BB*	13.45 (0.45)	11.91 (0.40)	1.13 (0.06)	0.69 (0.06)	0.37 (0.04)	9.32 (0.29)
	*p*-value	0.586	0.504	0.693	0.767	0.191	0.782

**PRRS**							

*Duroc*							
ASGA0031860	*AA*	7.91 (0.85)	5.27 (0.86)	1.62^B^ (0.19)	0.41 (0.15)	1.19 (0.18)	2.97 (0.83)
	*AB*	8.86 (0.43)	5.91 (0.43)	1.75^B^ (0.10)	0.70 (0.07)	0.93 (0.09)	3.81 (0.44)
	*BB*	8.90 (0.35)	5.43 (0.36)	2.26^A^ (0.08)	0.80 (0.06)	1.24 (0.08)	3.41 (0.37)
	*p*-value	0.458	0.300	0.058	0.175	0.120	0.317
MARC0089437	*AA*	8.35 (0.84)	5.36 (0.85)	1.92 (0.19)	0.40 (0.14)	1.42 (0.18)	3.70 (0.83)
	*AB*	8.19 (0.59)	5.36 (0.59)	1.62 (0.14)	0.69 (0.10)	0.83 (0.13)	3.52 (0.59)
	*BB*	9.12 (0.81)	5.89 (0.82)	2.07 (0.18)	0.84 (0.14)	1.14 (0.18)	2.97 (0.81)
	*p*-value	0.505	0.805	0.625	0.471	0.312	0.778
ASGA0032063	*AA*	8.34 (0.38)	5.44 (0.38)	1.74 (0.09)	0.62 (0.07)	1.09 (0.08)	3.61 (0.40)
	*AB*	8.77 (0.60)	5.63 (0.61)	2.00 (0.13)	0.64 (0.10)	1.14 (0.13)	3.18 (0.60)
	*p*-value	0.389	0.704	0.418	0.851	0.829	0.377
MARC0058875	*AA*	8.71 (0.77)	5.70 (0.77)	1.72 (0.17)	0.40 (0.13)	1.20 (0.16)	3.75 (0.77)
	*AB*	9.09 (0.62)	5.92 (0.63)	2.18 (0.14)	0.53 (0.11)	1.56 (0.14)	3.47 (0.62)
	*BB*	7.87 (0.85)	5.00 (0.86)	1.72 (0.19)	1.02 (0.15)	0.69 (0.19)	2.96 (0.84)
	*p*-value	0.425	0.638	0.558	0.239	0.131	0.825
ASGA0032151	*AA*	9.36 (0.72)	6.26 (0.73)	1.73 (0.16)	0.51 (0.12)	1.13 (0.15)	3.74 (0.71)
	*AB*	8.43 (0.50)	5.50 (0.51)	1.76 (0.11)	0.60 (0.09)	1.07 (0.11)	3.18 (0.52)
	*BB*	7.88 (0.51)	4.85 (0.51)	2.12 (0.12)	0.79 (0.09)	1.15 (0.11)	3.27 (0.52)
	*p*-value	0.209	0.232	0.567	0.387	0.924	0.675
*Landrace*							
ASGA0031860	*AA*	13.54 (0.77)	7.69 (0.89)	3.86 (0.16)	1.07 (0.12)	2.64 (0.17)	4.68 (0.72)
	*AB*	13.18 (0.49)	7.79 (0.56)	3.68 (0.10)	1.19 (0.08)	2.09 (0.11)	5.06 (0.46)
	*BB*	13.31 (0.47)	7.22 (0.54)	4.01 (0.10)	1.16 (0.08)	2.31 (0.10)	4.98 (0.45)
	*p*-value	0.853	0.622	0.793	0.860	0.457	0.834
MARC0089437	*AB*	13.69 (0.75)	7.78 (0.86)	3.74 (0.16)	1.18 (0.12)	2.20 (0.16)	5.16 (0.70)
	*BB*	13.00 (0.33)	7.35 (0.38)	3.96 (0.07)	1.10 (0.05)	2.48 (0.07)	4.65 (0.33)
	*p*-value	0.370	0.625	0.777	0.759	0.624	0.465
ASGA0032063	*AA*	14.30 (0.94)	8.09 (1.09)	4.74 (0.20)	1.18 (0.15)	2.86 (0.21)	4.88 (0.87)
	*AB*	12.98 (0.56)	7.26 (0.63)	3.73 (0.11)	1.16 (0.09)	2.41 (0.12)	4.86 (0.53)
	*BB*	12.76 (0.71)	7.35 (0.82)	3.20 (0.15)	1.09 (0.11)	1.82 (0.15)	4.98 (0.67)
	*p*-value	0.465	0.790	0.523	0.959	0.410	0.979
MARC0058875	*AA*	12.62 (0.63)	7.07 (0.73)	3.79 (0.13)	1.22 (0.10)	2.17 (0.14)	4.76 (0.60)
	*AB*	13.59 (0.52)	7.60 (0.60)	4.03 (0.11)	1.07 (0.08)	2.48 (0.11)	4.99 (0.49)
	*BB*	13.83 (0.73)	8.04 (0.85)	3.72 (0.15)	1.14 (0.12)	2.37 (0.16)	4.97 (0.69)
	*p*-value	0.402	0.700	0.838	0.793	0.816	0.943
ASGA0032151	*AA*	13.02 (0.63)	7.90 (0.73)	3.55 (0.13)	1.12 (0.10)	2.23 (0.14)	4.79 (0.59)
	*AB*	13.40 (0.53)	7.72 (0.61)	3.82 (0.11)	1.11 (0.08)	2.26 (0.12)	4.97 (0.51)
	*BB*	13.62 (0.62)	7.08 (0.72)	4.19 (0.13)	1.20 (0.10)	2.53 (0.14)	4.95 (0.59)
	*p*-value	0.736	0.691	0.766	0.928	0.866	0.944

**Post-PRRS**							

*Duroc*							
ASGA0031860	*AA*	9.36 (0.64)	8.26 (0.59)	0.73 (0.11)	0.59 (0.09)	0.16 (0.07)	7.87^A^ (0.60)
	*AB*	8.91 (0.30)	7.88 (0.28)	0.72 (0.05)	0.49 (0.04)	0.20 (0.03)	6.89^B^ (0.31)
	*BB*	8.94 (0.23)	7.89 (0.22)	0.68 (0.04)	0.44 (0.03)	0.23 (0.02)	6.67^B^ (0.25)
	*p*-value	0.741	0.777	0.822	0.378	0.599	0.090
MARC0089437	*AA*	10.03^a^ (0.62)	8.43^A^ (0.57)	1.02 (0.10)	0.58 (0.09)	0.44^a^ (0.07)	7.80^A^ (0.58)
	*AB*	9.32^a^ (0.43)	8.43^A^ (0.40)	0.60 (0.07)	0.46 (0.06)	0.12^b^ (0.05)	7.46^A^ (0.42)
	*BB*	7.85^b^ (0.57)	7.17^B^ (0.51)	0.55 (0.09)	0.48 (0.08)	0.05^b^ (0.06)	6.17^B^ (0.53)
	*p*-value	0.041	0.068	0.126	0.675	0.004	0.063
ASGA0032063	*AA*	8.77 (0.26)	7.78 (0.24)	0.68 (0.04)	0.51 (0.04)	0.16 (0.03)	6.88 (0.27)
	*AB*	9.36 (0.44)	8.23 (0.41)	0.74 (0.07)	0.49 (0.06)	0.23 (0.05)	7.40 (0.43)
	*p*-value	0.109	0.179	0.585	0.783	0.115	0.124
MARC0058875	*AA*	10.29^a^ (0.62)	8.82 (0.56)	0.89 (0.10)	0.56 (0.08)	0.33^a^ (0.07)	8.14^A^ (0.57)
	*AB*	8.89^ab^ (0.41)	7.81 (0.38)	0.72 (0.07)	0.46 (0.06)	0.26^a^ (0.04)	7.01^AB^ (0.40)
	*BB*	8.02^b^ (0.57)	7.40 (0.52)	0.54 (0.09)	0.49 (0.08)	0.02^b^ (0.06)	6.28^B^ (0.53)
	*p*-value	0.037	0.149	0.452	0.720	0.013	0.069
ASGA0032151	*AA*	8.92 (0.53)	7.95 (0.49)	0.69 (0.09)	0.49 (0.07)	0.19 (0.06)	7.22 (0.50)
	*AB*	9.14 (0.36)	7.98 (0.34)	0.76 (0.06)	0.55 (0.05)	0.18 (0.04)	7.09 (0.36)
	*BB*	9.15 (0.35)	8.10 (0.32)	0.69 (0.06)	0.47 (0.05)	0.22 (0.04)	7.12 (0.35)
	*p*-value	0.904	0.934	0.713	0.423	0.623	0.950
*Landrace*							
ASGA0031860	*AA*	14.61 (0.53)	13.16 (0.49)	0.97 (0.08)	0.53 (0.07)	0.39 (0.06)	10.48^a^ (0.45)
	*AB*	14.02 (0.35)	12.71 (0.33)	0.84 (0.05)	0.55 (0.05)	0.26 (0.04)	10.14^a^ (0.32)
	*BB*	13.58 (0.37)	12.35 (0.34)	0.84 (0.06)	0.49 (0.05)	0.32 (0.04)	9.45^b^ (0.33)
	*p*-value	0.221	0.320	0.676	0.657	0.133	0.050
MARC0089437	*AB*	14.54^A^ (0.53)	13.12 (0.49)	0.96 (0.08)	0.54 (0.07)	0.35 (0.06)	10.36 (0.46)
	*BB*	13.60^B^ (0.24)	12.35 (0.23)	0.82 (0.04)	0.50 (0.03)	0.30 (0.03)	9.69 (0.23)
	*p*-value	0.081	0.117	0.380	0.668	0.487	0.138
ASGA0032063	*AA*	14.13 (0.73)	13.14 (0.67)	0.65 (0.12)	0.34 (0.11)	0.29 (0.08)	10.21 (0.62)
	*AB*	14.06 (0.41)	12.53 (0.38)	0.98 (0.06)	0.60 (0.06)	0.32 (0.04)	9.78 (0.37)
	*BB*	14.03 (0.47)	12.55 (0.43)	1.05 (0.07)	0.63 (0.07)	0.36 (0.05)	10.09 (0.41)
	*p*-value	0.995	0.714	0.311	0.254	0.865	0.626
MARC0058875	*AA*	13.78 (0.47)	12.46 (0.44)	0.90 (0.07)	0.52 (0.07)	0.35 (0.05)	9.86 (0.41)
	*AB*	14.28 (0.37)	13.03 (0.35)	0.85 (0.06)	0.51 (0.06)	0.31 (0.04)	10.33 (0.33)
	*BB*	14.15 (0.54)	12.73 (0.49)	0.94 (0.08)	0.53 (0.08)	0.32 (0.06)	9.88 (0.46)
	*p*-value	0.626	0.398	0.840	0.974	0.883	0.269
ASGA0032151	*AA*	14.39 (0.47)	13.09 (0.44)	0.86 (0.07)	0.49^AB^ (0.07)	0.32 (0.05)	10.67^A^ (0.41)
	*AB*	14.07 (0.38)	12.82 (0.35)	0.85 (0.06)	0.43^B^ (0.06)	0.40 (0.04)	9.88^B^ (0.34)
	*BB*	13.74 (0.49)	12.30 (0.45)	0.94 (0.08)	0.65^A^ (0.07)	0.26 (0.05)	9.53^B^ (0.42)
	*p*-value	0.623	0.448	0.852	0.089	0.129	0.057

**TNB*, total number of piglets born; *NBA*, number of piglets born alive; *NBD*, number of piglets born dead; *NSB*, number of stillborn piglets; *NBM*, number of mummified piglets; *NW*, number of piglets weaned; *PRRS*, porcine reproductive and respiratory syndrome.*

*^1^Results for each genotype expressed as number of piglets (standard errors within parenthesis).*

*^2^SNPs are located on *Sus scrofa* chromosome 7 as follow according to the *Sscrofa11.1* (GCA_000003025.6) assembly: ASGA0031860/rs80959936 (7:22,075,114), MARC0089437/rs80900036 (7:24,217,931), ASGA0032063/rs80940999 (7:24,247,099), MARC0058875/rs80986722 (7:24,865,378), and ASGA0032151/rs80947467 (7:25,967,157). SNP markers MARC0089437 and ASGA0032063 were associated with S/P ratio in this study, in Duroc and Landrace populations, respectively. The other markers were associated with S/P ratio in Landrace sows in Serão et al. (2014).*

*^3^Results for NBD, NSB, and NBM were back-transformed from ln(phenotype+1).*

*^*a*–*b*^Expected values within row lacking the same superscript indicate differences at *p*-value <0.05.*

*^*A*–*B*^Expected values within row lacking the same superscript indicate differences at *p*-value <0.10.*

### Genomic Prediction

**FIGURE 3:**
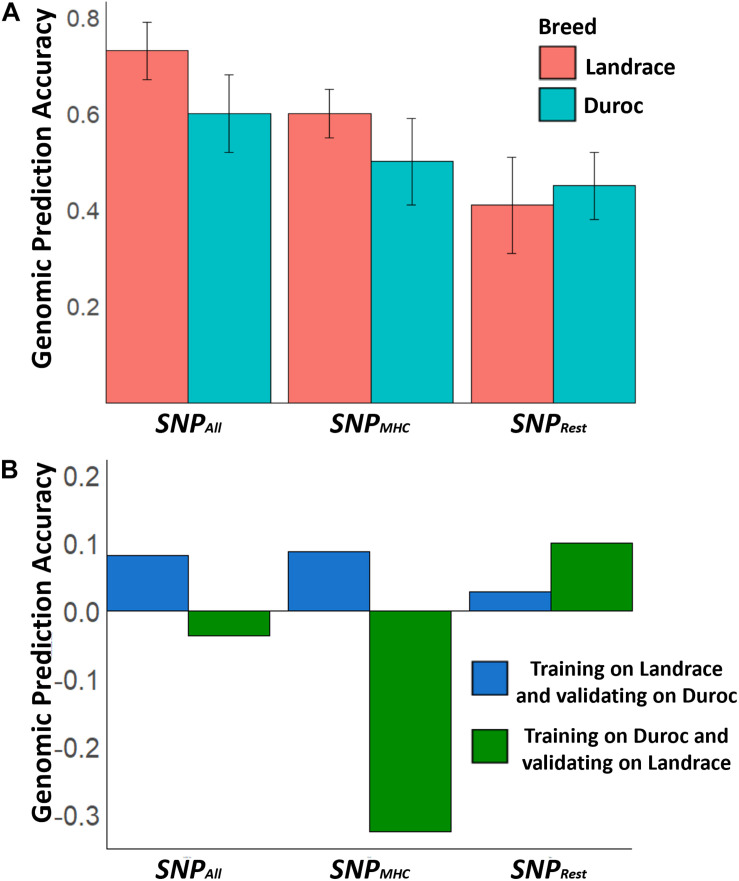
Genomic prediction accuracies of sample-to-positive (S/P) ratio across different SNP sets. **(A,B)** Genomic prediction accuracies for the within-breed and between-breed genomic prediction, respectively. SNP_All_ represents the set of SNPs using all 29,799 SNPs across the genome, while SNP_MHC_ accounts for only SNPs in the QTL that harbors the major histocompatibility complex (MHC) region. For SNP_Rest_, all SNPs across the genome were used excluding those in the MHC region and a 2-Mb window surrounding the QTL in the MHC region to avoid having any SNPs in linkage disequilibrium with this QTL. The error bars in panel **(A)** represent the standard deviations across the 5-fold used to calculate genomic prediction accuracies.

Genomic prediction accuracies (GPA ± standard deviation) were moderate to high for the within-breed analyses (Figure 3), showing that genomic selection for increased S/P ratio is feasible. GPAs were moderate to high, with greater values for SNP_All_, then SNP_MHC_, and then SNP_Rest_. These GPAs were 0.73 ± 0.06 for Landrace and 0.60 ± 0.08 for Duroc (SNP_All_), 0.60 ± 0.05 for Landrace and 0.50 ± 0.09 for Duroc (SNP_MHC_), and 0.41 ± 0.10 for Landrace and 0.45 ± 0.07 for Duroc (SNP_Rest_), indicating that genomic selection for S/P ratio, regardless of the genomic information used, is feasible. On the other hand, for the between-breed analysis, GPAs were low and sometimes negative. For SNP_All_, SNP_MHC_, and SNP_Rest_, these were −0.03, −0.32, and 0.10, respectively, when training on Duroc and validating on Landrace, and 0.08, 0.09, and 0.03, respectively, when training on Landrace and validating on Duroc. These results indicate that between-breed genomic selection has limited usefulness.

## Discussion

### Genetic Parameters

Heritability estimates of S/P ratio were overall lower than in Serão et al. (2014), who reported a high heritability estimate (0.45 ± 0.13) for S/P ratio following a PRRS outbreak in purebred Landrace sows. There were differences in collection dates between our study and Serão et al. (2014). While in Serão et al. (2014), blood samples were taken from purebred Landrace sows at approximately 46 days after the estimated beginning of the PRRS outbreak, in our study it was at about 54 days. Thus, this difference in the collection dates between both studies might have affected results since antibody response is time sensitive. Differences in estimates could also be due to differences in genetic background between populations, random variation in the estimation of the parameters, or inaccuracy in identifying the PRRS outbreak period.

Other studies have also estimated the heritability of S/P ratio. Serão et al. (2016) reported a high heritability of S/P ratio (0.47) measured at an average of 40.8 days (SD = 16.3) after F1 gilts entered the commercial farm (no confirmation on whether vaccination or PRRSV wild-type infection was obtained in their study). Using PRRSV-infected purebred sows, Putz et al. (2019) reported a heritability estimate of 0.17 for S/P ratio measured at about 60 days after the outbreak. However, these authors did not use the same method to measure S/P ratio as in our study. Abella et al. (2019) and Sanglard et al. (2020) reported heritability estimates of 0.69 and 0.33 for S/P ratio in F1 gilts after vaccinating animals with a modified live PRRSV vaccine (MLV) at 42 and 52 days, respectively. Although there are major differences between these estimates, S/P ratio was measured in young gilts in Abella et al. (2019), with 6–7 weeks of age, whereas S/P ratio was measured in more mature gilts in Sanglard et al. (2020) with 26 weeks of age. Therefore, differences in heritability estimates available in the literature may be due to several factors, including the time of collection, the method used to measure S/P ratio, the age of the animals, type of exposure (vaccination or natural infection), and random error. Our results further support the idea that S/P ratio has a sizeable genetic component.

Estimates of genetic correlations of S/P ratio with litter size traits during the PRRS outbreak were, overall, consistent with the results of Serão et al. (2014) for Landrace sows. This is the first study reporting results using a terminal sire line (i.e., Duroc sows) to the best of our knowledge. Serão et al. (2014) reported that S/P ratio had strong favorable genetic correlations with NSB (−0.72 ± 0.28) and NBA (0.73 ± 24) during a PRRS outbreak. In our study, there was also a high favorable genetic correlation of S/P ratio with NBA (0.61 ± 0.34), but not for NSB (−0.27 ± 0.31). We also found a favorable genetic correlation of S/P ratio with TNB (0.47 ± 1.47) in Landrace sows. However, this estimate had a large standard error, probably due to the low heritability estimate of TNB (0.01 ± 0.05) and the small sample size. The genetic correlations of S/P ratio with mortality traits were in a favorable direction (negative) but not as strong (≤ −0.33) as results from Serão et al. (2014). Putz et al. (2019) only found a large genetic correlation of S/P ratio with NSB (−0.73 ± 0.29), in contrast to our study. These results indicate that S/P ratio can be used as an indicator trait for improved reproductive performance in Landrace sows during a PRRS outbreak.

**FIGURE 4:**
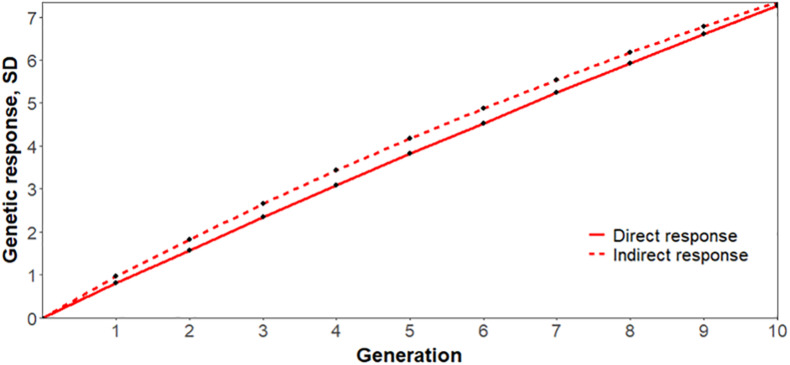
Simulated response to selection for increased number of piglets born alive (NBA) in Landrace sows after 10 generations based on (indirect) or not (direct) antibody response to porcine reproductive and respiratory syndrome virus infection, measured as sample-to-positive (S/P) ratio. The *y*- and *x*-axis represent the response to selection in genetic standard deviations and generations, respectively. Direct and indirect response to selection are represented by solid and dashed lines, respectively, assuming 5% selection intensity, using the genetic parameters obtained in this study.

As reported in Serão et al. (2014), an indirect response to selection on reproductive performance during a PRRS outbreak based on S/P ratio is expected to be 63% more effective than a direct response to selection for increased NBA during a PRRS outbreak. Based on our genetic parameters, the corresponding estimate would be 22%, further supporting the use of S/P ratio as an indicator trait for reproductive performance under a PRRSV infection, as illustrated in Figure 4. It is important to note that this is a simplistic comparison, assuming that selection is performed on own phenotypes. Results for Duroc were not as promising. In addition to having lower estimates of genetic correlation of S/P ratio with reproductive traits, we had convergence issues for genetic correlations with mortality traits.

Under the conditions described in this study, Serão et al. (2014), and Putz et al. (2019), the use of S/P ratio as a genetic indicator trait for improved reproductive performance under a PRRS outbreak has some limitations. For instance, these studies reported a high genetic correlation of S/P ratio with and reproductive performance using (i) purebred animals (ii) under a natural PRRS outbreak. In practice, breeding companies do not want to have PRRSV-positive purebred animals, as this can impact the additive genetic response to other traits measured in these animals that are included in their breeding goals, impacting the actual meaning of their (G)EBVs, as well as limiting the use of purebred animals for breeding. In addition, it is not expected that purebred herds undergo a PRRS outbreak due to the high biosecurity in these hers. However, they can happen in practice, such as for the populations used in this and other studies. Furthermore, although several studies have demonstrated that, in PRRSV-infected purebred sows, S/P ratio has sizable heritability and genetic correlation with reproductive performance, a large number of animals is needed to obtain accurate (G)EBVs for animals to be selected based on S/P ratio to improve reproductive performance under a PRRS outbreak. Finally, the overall goal of purebred selection is to improve the performance of crossbred animals, which was not evaluated in this study. Hence, the applicability of such a tool under these scenarios is limited. Nonetheless, the validation of the results reported by Serão et al. (2014) brings new opportunities to develop and evaluate feasible strategies to use S/P ratio as an indicator trait.

For instance, based on the results reported by Abella et al. (2019) and Sanglard et al. (2020), potential strategies are feasible. These authors reported that their studies were driven by the results reported by Serão et al. (2014) and proposed evaluating the use of S/P ratio as an indicator trait for reproductive performance in PRRSV-vaccinated F1 gilts. These authors reported moderate-to-high heritability estimates of S/P ratio (0.69 and 0.33, respectively). Furthermore, Sanglard et al. (2020) showed that S/P ratio due to vaccination was highly genetically correlated with NBA in F1 animals, while no PRRS outbreak was present during pregnancy or farrowing of these animals. Although the use of PRRSV vaccination is a standard procedure in the U.S. swine industry (Arruda et al., 2016), this strategy requires a close genetic relationship between F1 animals and nucleus animals. For example, F1 animals should have pedigree information available to estimate the breeding values of nucleus animals for S/P ratio and reproductive performance. Another strategy is to genotype F1 animals, which is desirable but still cost limiting. Nonetheless, investigating the relationships among S/P ratio and reproductive performance, under or not PRRSV infection or vaccination, is needed to further evaluate this strategy.

Sanglard et al. (2021) combined the S/P ratio data from the PRRSV-vaccinated F1 animals in Sanglard et al. (2020) and the Landrace population used in this study to investigate the genetic relationships between S/P ratio and reproductive performance under several conditions. These authors reported that S/P ratio due to vaccination in F1 gilts and PRRSV infection in Landrace nucleus sows was high (0.72), indicating that the host genetic response to PRRS challenge is similar. Furthermore, these authors reported a favorable and moderate genetic correlation (0.50) of S/P ratio in PRRSV-vaccinated gilts with NBA in nucleus animals before the PRRS outbreak. However, contrary to our expectations, the estimate between S/P ratio in PRRSV-vaccinated gilts and NBA in purebred Landrace during the PRRS outbreak was close to zero (0.07). Nonetheless, when this relationship was evaluated between S/P ratio in PRRSV-infected Landrace and NBA in F1 gilts, a favorable, albeit low, estimate of genetic correlation was observed (0.23). Hence, the use of S/P ratio data collected in Landrace nucleus herds during a PRRS outbreak could be used to predict reproductive performance in commercial F1 animals. Finally, Hickmann et al. (2021), using the same animals in this study, reported high genetic correlation estimates for reproductive performance before and during the PRRS outbreak. Therefore, combining the results from Hickmann et al. (2021), Sanglard et al. (2021), and the current study, the use of S/P ratio, either from collecting data in PRRSV-infected nucleus Landrace animals or PRRSV-infected F1 gilts, as an indicator trait for reproductive performance (under or not PRRSV infection) is possible.

In summary, antibody response to PRRSV, measured as S/P ratio, was shown to be moderately heritable in Landrace and Duroc sows during a PRRS outbreak. In combination with the high genetic correlation of S/P ratio with NBA in Landrace (0.61) and the negative genetic correlations with mortality traits, our results validate and further support the use of S/P ratio as an indicator trait for improved reproductive performance under a PRRS outbreak in Landrace populations. However, in Duroc, the weak genetic correlation estimates and the large standard errors do not allow us to make conclusions for this population.

### Genomic Regions in the Pig Genome Associated With S/P Ratio

In this study, the QTL for S/P ratio identified on SSC7 (24–25 Mb) for S/P ratio in Duroc and Landrace sows is located within the MHC region, as previously reported by Serão et al. (2014, 2016) and Sanglard et al. (2020). The MHC region is widely recognized as the most important genomic region controlling the immune response in mammals (Hammer et al., 2020). The MHC QTL explained 25% of TGVM for S/P ratio in Landrace sows in Serão et al. (2014) and 20% of TGVM for S/P ratio in F1 replacement gilts in Serão et al. (2016). In our study, this QTL explained a greater proportion of TGVM in Landrace (31%) but lower in Duroc (15%). Their genetic background could partially explain the difference between these breeds since Landrace pigs have been intensively selected for a different set of traits (i.e., maternal traits) than Duroc pigs (i.e., terminal traits). In addition, it could be that the LD between SNPs and QTL in this region may differ between the two populations, affecting the power to detect the QTL.

Previous studies also reported a QTL for S/P ratio at 128–132 Mb on SSC7 that explained 15% (Serão et al., 2014) and 7% (Serão et al., 2016) of TGVM. In these reports, the older version of the swine genome assembly was used (i.e., *Sscrofa10.2*). The QTL identified by these authors around 130 Mb on the draft genome assembly (*Sscrofa10.2*) was not identified in our study, but these authors indicated that this region shows high LD. However, there are some errors in the *Sscrofa11.1* assembly in the part of SSC 7 corresponding to 128–132 Mb in the *Sscrof10.2* assembly, with most of the missing content now located on an unplaced scaffold (AEMK02000452) in the *Sscrofa11.1* assembly (Warr et al., 2020). Nonetheless, a GWAS using the older assembly version still did not identify this QTL in our data (results not shown). Interestingly, Sanglard et al. (2020), using PRRSV-vaccinated F1 gilts genetically related to the Landrace animals used in this study, also did not find this QTL in their analyses of S/P ratio, while they did identify the MHC QTL. Thus, the reason why the 130-Mb QTL on SSC7 detected by Serão et al. (2014, 2016) was not detected by Sanglard et al. (2020) and in the current study could be because this QTL is segregating in our population or due to the lack of LD between SNPs and the QTL. Serão et al. (2016) detected this QTL using data from seven breeding companies. However, analyses performed by each breeding company did not identify this QTL for all companies (unpublished results), further suggesting that this QTL may not be segregating or lack SNP-QTL LD in all populations.

We also found novel QTL on SSC8 (25 Mb) in Duroc and on SSC7 (108–109 Mb) in Landrace sows. However, they explained a much smaller proportion of TGVM, 2.4 and 2.2%, respectively. The QTL at 108–109 Mb on SSC 7 had two candidate genes associated with reproduction or immune response. The G protein-coupled receptor 65 (*GPR65*), a protein-coding gene that has been associated with immune response in humans by regulating the cytokine production of T cells and macrophages (Onozama et al., 2012), and the *GALC* gene, which has been associated with spermiogenesis and with sperm abnormalities in mouse when deficient (Luddi et al., 2005). These candidate genes further support that this region may play a role in immune response and reproduction. No candidate genes were identified for the SSC8 QTL found for Duroc.

### Effect of Selected SNPs Associated With S/P Ratio

We performed additional analyses using the SNPs associated with S/P ratio that accounted for most of the TGVM observed in our study, along with those reported by Serão et al. (2014). These authors evaluated the effect of SNPs on S/P ratio measured in Landrace sows that experienced a PRRS outbreak. They reported five SNPs within the MHC QTL found in their study (SSC7, 24–30 Mb) that accounted for 25.1% of TGVM. Of their SNPs, three of them are located within the MHC QTL in our study: ASGA0032151, ASGA0031860, and MARC0058875. In Serão et al. (2014), the ASGA0032151 and MARC0058875 SNPs were associated with S/P ratio, with the *AB* and *BB* genotypes having a greater S/P ratio than the *AA* genotype at both SNPs.

In Duroc sows, the MHC QTL that explained 15% of TGVM explained only 1.5% of TGVM after removing these five SNPs from the MHC QTL, indicating that these SNPs accounted for most of the effect in the MHC region. Similarly, for Landrace sows, the MHC QTL that explained 31% of TGVM explained < 0.1% of TGVM after these five SNPs were accounted for. This indicates that these SNPs were capable of accounting for the TGVM of S/P ratio within this region. The MARC0089437 SNP was the only SNP that was associated with S/P ratio in both populations. Interestingly, this SNP was only identified as a key SNP in the GWAS using Duroc sows.

Furthermore, the direction of the effects for this SNP was opposite in the two populations. Although a greater S/P ratio was obtained in Duroc sows with the *AB* and *BB* genotypes, Landrace sows with the *BB* genotype had a lower S/P ratio than *AB* sows. Also, the A allele had a very low frequency (0.04) in Landrace sows but a much higher frequency (0.41) in Duroc. In fact, only one Landrace sow had the *AA* genotype, and this individual was removed from the analysis. We found a similar situation for the ASGA0032063 SNP identified in the GWAS for Landrace, for which the B allele had a very low frequency (0.05) in Duroc sows, but a much greater frequency (0.69) in Landrace sows. Heterozygote Landrace sows had a greater S/P ratio than homozygote sows, indicating an overdominance effect for this SNP. This has some limitations in breeding schemes in the nucleus, as selection for improved performance results in fixation of the favorable allele, limiting the number of *AB* animals for this SNP. Nonetheless, *AA* Landrace sows had a greater S/P ratio than *BB* sows for this SNP, which indicates that selection for an increase in the frequency of the A allele in purebred Landrace sows may increase the S/P ratio of the population.

For the SNPs selected based on the results of Serão et al. (2014), MARC0058875 was associated with S/P ratio in Duroc sows and ASGA0032151 in Landrace sows during the PRRS outbreak. Duroc sows with the *AB* genotype at the MARC0058875 SNP had a lower S/P ratio than homozygous animals, indicating a negative dominance effect for this SNP. Serão et al. (2014), using Landrace sows, observed the superiority of *AB* and *BB* compared with *AA* sows at the MARC0058875 SNP. This contrasting result indicates a complex relationship between this marker and the QTL in these distinct populations. For ASGA0032151, the *BB* genotype had a greater S/P ratio than *AA* in Landrace animals. This is partially in accordance with Serão et al. (2014), who also worked with Landrace animals during a PRRS outbreak. However, these authors also observed a greater S/P ratio in *BB* sows than *AB* genotypes, whereas no differences in S/P ratio were observed in our study between *AB* and *BB* sows. In addition, the B allele was highly frequent in both populations, with 0.46 and 0.41 in Serão et al. (2014) and in our study, respectively, indicating that selection for increased frequency of this favorable allele may be performed with regards to S/P ratio. These results bring new possibilities for marker-assisted selection for greater antibody response in PRRSV-infected sows.

### Effects of Selected SNPs Associated With S/P Ration on Reproductive Traits

Given the hypothesis of S/P ratio being an indicator trait for reproductive performance in PRRSV-infected (Serão et al., 2014) and healthy sows (Sanglard et al., 2020), we evaluated the effects of the five selected SNPs associated with S/P ratio from Serão et al. (2014) and from the current study on reproductive performance before, during, and after the PRRS outbreak. Although the MHC region was not associated with any of the reproductive traits evaluated in a study using the same animals (Hickmann et al., 2021), the genetic variation in the MHC region has been associated with reproductive performance in other studies (Vaiman et al., 1998; Laplana et al., 2020; Sanglard et al., 2020). Sanglard et al. (2020) reported a SNP associated with S/P ratio in PRRSV-vaccinated gilts that was also associated with reproductive performance in the absence of PRRSV infection, even if this SNP was not detected in the univariate GWAS for reproductive traits.

In Duroc sows, only the MARC0089437 and MARC0058875 SNPs were associated with S/P ratio. However, they were associated with reproductive traits only outside the PRRS phase. At the MARC0089437 SNP, a greater S/P ratio was obtained for the *AB* and *BB* genotypes. For reproductive traits, *AB* sows had greater NBA pre-PRRS and TNB, NBA, and NW post-PRRS for this SNP. These results indicate that selection for heterozygotes at the MARC0089437 SNP may increase not only S/P ratio but also TNB, NBA, and NW in sows not facing a PRRS outbreak. At the MARC0058875 SNP, *AA* and *AB* animals had greater S/P ratio than *AB* animals. Although there was under-dominance for this SNP for S/P ratio, *AA* animals had greater TNB and NW post-PRRS, indicating that selection for fixation of the A allele would result in animals with greater post-PRRS reproductive performance and S/P ratio. Although not significantly associated with TNB and NW in the pre-PRRS phase, the *AA* genotype showed numerically greater performance than the other genotypes, suggesting that fixation of the favorable allele for this SNP might increase performance even in PRRSV-naïve animals. This is important as it is expected that purebred animals in the nucleus will not go through a PRRS outbreak. Hence, significant associations between the MARC0058875 SNP and pre-PRRS performance would further suggest that this SNP might be important in explaining variation in reproductive performance of purebred sows even in the absence of a PRRS outbreak. Interestingly, the only selected SNP associated with reproductive performance during the PRRS phase was ASGA0031860. Although this SNP was not associated with S/P ratio in Duroc pigs, greater NBD was obtained in *BB* animals.

It is worth noting that, based on estimates of genetic correlations, we suggested that S/P ratio might not be an indicator trait for reproductive performance in Duroc pigs. However, the two SNPs associated with S/P ratio in Duroc sows (MARC0089437 and MARC0058875) were also associated with some reproductive traits for this breed, with all favorable genotypes being overall the same for S/P ratio and reproductive traits. Thus, although we could not find strong genetic correlations between S/P ratio and reproductive traits, it could be that these SNPs are capturing pleiotropic QTL(s) for these traits, similar to what Sanglard et al. (2020) reported. In contrast, the ASGA0031860 SNP identified by Serão et al. (2014) was not associated with S/P ratio in Duroc, but it was associated with NBD during the PRRS phase in our study. For this association, it could be that a reproduction-specific QTL in the MHC region is captured by this SNP, as this region has been associated with reproduction in other studies using healthy pigs (Vaiman et al., 1998). Nonetheless, it could also be that these associations are spurious as the sample size of this study is relatively small for obtaining accurate estimates for the genetic correlation of S/P ratio with reproductive traits. Hence, it could also be that S/P ratio and reproductive traits share a common genomic basis, such as observed in Landrace sows, which would indicate that these associations between selected SNPs for S/P ratio and reproductive traits in Duroc pigs are real. However, the statistical power to obtain these estimates was low, resulting in weak estimates of genetic correlations between S/P ratio and reproductive traits in Duroc pigs in our study, given the large standard errors. Nonetheless, additional studies are needed to better investigate the effect of these SNPs on reproductive traits in Duroc sows.

For Landrace, the three SNPs associated with S/P ratio were ASGA0032063, ASGA0032151, and MARC0089437. However, these SNPs were only associated with reproductive performance in the absence of PRRS. At the ASGA0032063 SNP, *AB* animals had a greater S/P ratio, followed by *AA* and then by *BB* animals. For pre-PRRS NBD and NBS, *BB* sows also had lower performance but not different than *AB* sows. Thus, these results suggest that greater reproductive performance might be obtained by increasing the frequency of the *AA* genotype at this SNP in the population. For S/P ratio, the ASGA0032151 SNP, *BB* sows had a greater S/P ratio than *AA* sows in our study, similar to the results of Serão et al. (2014), who first reported the association between this SNP and S/P ratio. Following what we found for S/P ratio, *AA* animals had better performance in post-PRRS NSB and NW than *BB* animals, indicating that selection for increased frequency of the A allele at this SNP would result in overall better improved reproductive performance. On the other hand, a greater S/P ratio was obtained in *AB* animals at the MARC0089437 SNP. Interestingly, although sows with this genotype had lower pre-PRRS NW (9.1 ± 0.33) than *BB* sows (9.7 ± 0.19), the same direction of effects for S/P ratio was observed in post-PRRS TNB, in which *AB* animals had greater performance than *BB* animals. Finally, although the ASGA0031860 SNP was not associated with S/P ratio in Landrace sows in our study, *AA* animals had greater post-PRRS NW than *BB* animals, bringing the possibility of selection for increased NW based on this SNP regardless of S/P ratio.

The lack of associations between these selected SNPs for S/P ratio and reproductive performance during the PRRS outbreak in this study was unexpected. Moreover, the fact that associations of S/P ratio SNPs with reproductive performance were only found in the pre-PRRS and post-PRRS phases could be considered contradictory to the proposed use of S/P ratio as an indicator trait during a PRRS outbreak. Therefore, these issues must be addressed. To begin with, previous studies have associated polymorphisms in the MHC region with reproductive performance in healthy animals (Vaiman et al., 1998; Laplana et al., 2020; Sanglard et al., 2020). Given that S/P ratio had moderate-high heritability estimates in our study and that this trait is not under selection, we expect to find key SNPs for this trait more easily than for reproductive traits, which are lowly heritable and under selection for decades. Therefore, assuming that the MHC has a true effect on reproductive performance, even if the MHC was not identified for these traits in this population (Hickmann et al., 2021), and these traits are genetically correlated with S/P ratio, we could expect to find associations between S/P ratio in the MHC with reproductive traits regardless of PRRS challenge. This could be even more evident for Duroc sows, in which the MARC0089437 SNP accounted for by 9.2% of TGVM for S/P ratio and associations between this SNP were found for reproductive traits in the pre-PRRS and post-PRRS phases, even if we did not observe evidence for S/P ratio to be used as an indicator trait for this breed.

The statistical power to detect these associations during the PRRS outbreak could be lower than in the absence of infection. The residual variance of traits under selections measured during challenge conditions is expected to be greater than in the lack of challenge (Berghof et al., 2019). In fact, the residual variances of the reproductive traits evaluated in this study were generally greater in the PRRS phase than in the other phases (Hickmann et al., 2021). Therefore, the power to detect SNP associations is expected to be lower in the PRRS phase than in the other phases, which could explain the lack of associations during the PRRS outbreak period.

The greater number of associations in the post-PRRS phase than in the others could be due to two factors. First, a greater number of sows and litters were used for analyses in the post-PRRS phase than the others (details in Hickmann et al., 2021), which could have increased the statistical power to detect significant associations between S/P ratio SNPs and reproductive performance. Second, these associations might be more powerful to be detected once animals have been challenged. This is in accordance with Sanglard et al. (2020). They reported significant associations between S/P ratio SNP and reproductive performance in sows that were not under a PRRS outbreak but had been PRRSV vaccinated when they were gilts. Hence, we hypothesize that the immune system of these animals must be activated at some level to identify associations between S/P ratio SNPs and reproductive performance with greater power.

Finally, the proposed use of S/P ratio as an indicator trait for reproductive performance during a PRRS outbreak is based on the moderate-high heritability of this trait and its genetic correlation with reproductive performance during PRRS. More importantly, these results validate the original proposal by Serão et al. (2014). The lack of S/P ratio SNP associations with reproductive performance under PRRS does not impact the novelty of S/P ratio due to the previous points raised in this section. This illustrates some of the challenges of performing genomic analyses for complex lowly heritable traits such as reproductive traits. Nonetheless, these results bring new possibilities for marker-assisted selection for greater antibody response and improved reproductive performance based on the selected SNPs reported in this study. However, a larger and independent dataset might be needed to identify significant associations between S/P ratio SNPs and reproductive performance across PRRS phases.

### Genomic Prediction Accuracies

Studies on genomic prediction of antibody response to PRRSV are scarce in the literature. To the best of our knowledge, Serão et al. (2016) is the only study that performed genomic prediction analyses for S/P ratio in sows during a PRRS outbreak. These authors reported GPAs using two strategies: using crossbred F1 gilts during acclimation for training and validation or using crossbred F1 gilts during acclimation for training and a purebred population under a PRRS outbreak (Serão et al., 2014) for validation. The same SNP set strategies used by Serão et al. (2016) (SNP_All_, SNP_MHC_, and SNP_Rest_) were used in our study. For their first strategy, the GPAs were 0.33, 0.24, and 0.09 for SNP_All_, SNP_MHC_, and SNP_Rest_, respectively. For their second strategy, these were 0.45, 0.40, and 0.10, respectively. These GPAs reported by Serão et al. (2016) were much lower than those from our within-breed genomic prediction analyses. This major discrepancy between results could be because Serão et al. (2016) performed analyses using data from seven breeding companies, where animals from the same breeding company were not simultaneously used in the training and validation dataset. In contrast, we used data from closely related animals, which took advantage of genetic similarities between the training and validation datasets, which is expected to increase GPAs. Sanglard et al. (2020) also used genetically related animals for the genomic prediction of S/P ratio in PRRSV-vaccinated F1 gilts that shared genetic relationships with the animals used in our study. These authors reported GPAs of 0.67, 0.59, and 0.34 for SNP_All_, SNP_MHC_, and SNP_Rest_, respectively, similar to those obtained in our study.

With regard to the between-breed scenario, our results were much worse than those in Serão et al. (2016) for SNP_All_ and SNP_MHC_, whereas the one for SNP_Rest_ when training in Duroc and validating in Landrace (GPA = 0.09) was very similar to the result found in Serão et al. (2016). In general, between-breed GPAs indicate that this strategy should not be used in genomic selection for S/P ratio. The only sizeable GPA was obtained for SNP_MHC_ when training in Duroc and validating in Landrace. Interestingly, we obtained a negative GPA (−0.32). However, we observed a small positive GPA for this SNP set when Landrace was used for training and Duroc for validation (GPA = 0.09). These two contrasting results could suggest a combination of events. For example, it could be that the LD between SNPs and a major QTL in the MHC for Duroc and Landrace are in opposite phases between the two breeds. Also, it could be that some of the QTL effects captured by SNPs using Duroc sows are not segregating in Landrace sows. Finally, when Landrace is used for training, SNPs could be capturing few small effects of QTL on the same phase in both breeds, explaining the small and positive GPA for this scenario, whereas this was not observed when training using Duroc pigs. Nonetheless, the between-breed results show that genomic selection for S/P ratio should not be performed across breeds based on our results. The high within-breed genomic prediction accuracies for S/P ratio indicate that genomic selection within a breed is an efficient strategy to change S/P ratio within Landrace and Duroc populations. However, the sample size used in this study was still limited to obtain very accurate estimates for the measures reported. Therefore, a larger sample size would be needed to validate these results.

## Conclusion

Antibody response to PRRSV infection, measured as S/P ratio, was shown to be moderately heritable in Landrace and Duroc sows following a PRRS outbreak. In combination with a high estimated genetic correlation of S/P ratio with NBA (0.61) in Landrace sows, our results validate and further support the use of S/P ratio as an indicator trait for improved reproductive performance during a PRRS outbreak. However, this seems to work only for Landrace populations. In Duroc, the weak estimates of genetic correlations of S/P ratio with reproductive performance and their large standard errors do not allow us to propose using S/P ratio as an indicator trait in this breed. Our genomic analyses further validated the major histocompatibility region as the major QTL for S/P ratio during a PRRS outbreak in Landrace, showing that this QTL also plays a major role for S/P ratio in Duroc pigs. In addition, we identified novel small-effect QTL on SSC7 (108–109 Mb) in Landrace and on SSC8 (25 Mb) in Duroc sows. We also provided information on specific SNPs within the major histocompatibility region in both populations, providing the opportunity of marker-assisted selection for increased S/P ratio and reproductive performance. Finally, the high genomic prediction accuracies for S/P ratio indicate that genomic selection within a breed is an efficient strategy to change S/P ratio in Landrace and Duroc populations.

## Data Availability Statement

The datasets presented in this article are not readily available because the data that support the findings of this study are not publicly available. Data may be available from authors upon reasonable request and authorization from the company that generated the data.

## Ethics Statement

The animal study was reviewed and approved by the Institutional Animal Care and Use Committee (IACUC) at Iowa State University (protocol number 6-17-8551-S). Written informed consent was obtained from the owners for the participation of their animals in this study.

## Author Contributions

FH performed statistical analyses, interpreted results, prepared figures and tables, and drafted the manuscript. JB, LK, and JD were involved in the interpretation and discussion of results. YH and KG provided the data, led the collection of blood samples, and interpreted results. LS provided help and guidance for statistical analyses, being involved in the discussion of results as well. NS assisted with data analysis, interpretation of results, and drafted the manuscript. All authors read and approved the final version of the manuscript.

## Conflict of Interest

The authors declare that this study received funding from Smithfield Premium Genetics, NC, United States. The funder had the following involvement with the study: providing performance and genotype data and collection of blood samples. In addition, YH and KG are employed by the company Smithfield Premium Genetics, NC, United States. The remaining authors declare that the research was conducted in the absence of any commercial or financial relationships that could be construed as a potential conflict of interest.

## Publisher’s Note

All claims expressed in this article are solely those of the authors and do not necessarily represent those of their affiliated organizations, or those of the publisher, the editors and the reviewers. Any product that may be evaluated in this article, or claim that may be made by its manufacturer, is not guaranteed or endorsed by the publisher.

## Abbreviations

Chr: chromosome
ELISA: enzyme-linked immunosorbent assay
GRM: genomic relationship matrix
GEBV: genomic estimated breeding value
GPA: genomic prediction accuracy
GWAS: genome-wide association studies
LD: linkage disequilibrium
Mb: megabases
MCMC: Markov Chain Monte Carlo
MHC: major histocompatibility complex
MLV: modified live PRRSV vaccine
NBA: number of piglets born alive
NBD: number of piglets born dead
NBM: number of mummified piglets
NSB: number of stillborn piglets
NW: number of piglets weaned
PRRS: porcine reproductive and respiratory syndrome
PRRSV: porcine reproductive and respiratory syndrome virus
QTL: quantitative trait loci
S/P ratio: sample-to-positive ratio
SNP: single nucleotide polymorphisms
SNP_All_: all SNPs across the genome
SNP_MHC_: only SNPs in the QTL that harbors the MHC region
SNP_Rest_, all SNPs across the genome: excluding those in the MHC region
SSC: *Sus scrofa* chromosome
TGVM: total genetic variance accounted for by markers
TNB: total number of piglets born.
